# A new class of insecticide for malaria vector control: evaluation of mosquito nets treated singly with indoxacarb (oxadiazine) or with a pyrethroid mixture against *Anopheles gambiae* and *Culex quinquefasciatus*

**DOI:** 10.1186/s12936-015-0890-1

**Published:** 2015-09-17

**Authors:** Richard M. Oxborough, Raphael N’Guessan, Jovin Kitau, Patrick K. Tungu, David Malone, Franklin W. Mosha, Mark W. Rowland

**Affiliations:** Department of Disease Control, London School of Hygiene and Tropical Medicine, London, UK; Department of Entomology and Parasitology, Kilimanjaro Christian Medical University College, Moshi, Kilimanjaro Tanzania; Laboratoire Nationale, Ministère de la Santé, CREC Laboratories, Centre de Recherche Entomologique de Cotonou, Cotonou 06, BP 2604, Benin; Pan-African Malaria Vector Research Consortium, (PAMVERC), Moshi, Tanzania; Amani Centre, National Institute for Medical Research, Muheza, Tanzania; Innovative Vector Control Consortium (IVCC), Pembroke Place, Liverpool, L3 5QA UK

**Keywords:** Indoxacarb, Oxadiazine, ITN, Insecticide mixtures, *Anopheles gambiae*, Pyrethroid resistance

## Abstract

**Background:**

Universal coverage with long-lasting insecticidal mosquito nets (LLIN) or indoor residual spraying (IRS) of houses remain the primary strategies for the control of mosquito vectors of malaria. Pyrethroid resistant malaria vectors are widespread throughout sub-Saharan Africa and new insecticides with different modes of action are urgently needed if malaria vector control is to remain effective. Indoxacarb is an oxadiazine insecticide that is effective as an oral and contact insecticide against a broad spectrum of agricultural pests and, due to its unique site of action, no cross-resistance has been detected through mechanisms associated with resistance to insecticides currently used in public health.

**Methods:**

WHO tunnel tests of host seeking mosquitoes were carried out as a forerunner to experimental hut trials, to provide information on dosage-dependent mortality, repellency, and blood-feeding inhibition. A dosage range of indoxacarb treated netting (100–1000 mg/m^2^) was tested against a pyrethroid susceptible strain of *Anopheles gambiae*. In addition, efficacy of indoxacarb 500 mg/m^2^ was compared with a standard pyrethroid formulation against pyrethroid susceptible and resistant *Culex quinquefasciatus*. Dosages between 25 and 300 mg/m^2^ indoxacarb were tested in tunnel tests and in ball-frame bioassays as mixtures with alphacypermethrin 25 mg/m^2^ and were compared with singly applied treatments against an insectary reared pyrethroid resistant strain of *Cx. quinquefasciatus* originally collected in Cotonou, Benin.

**Results:**

There was a dosage-dependent response in terms of indoxacarb induced mortality, with dosages >100 mg/m^2^ producing the best mortality response. In tunnel tests indoxacarb 500 mg/m^2^ exceeded WHOPES thresholds with >80 % mortality of adult *An. gambiae* and blood-feeding inhibition of 75 %. No cross-resistance to indoxacarb was detected through mechanisms associated with resistance to pyrethroid insecticides and was equally effective against susceptible and resistant strains of *Cx. quinquefasciatus*. Indoxacarb 500 mg/m^2^ killed 75 % of pyrethroid resistant *Cx. quinquefasciatus* compared with only 21 % mortality with alphacypermethrin 40 mg/m^2^. Mixtures of indoxacarb with pyrethroid produced an additive response for both mortality and blood-feeding inhibition. The best performing mixture (indoxacarb 200 mg/m^2^ + alphacypermethrin 25 mg/m^2^) killed 83 % of pyrethroid resistant *Cx. quinquefasciatus* and reduced blood-feeding by 88 %, while alphacypermethrin only killed 36 % and inhibited blood-feeding by 50 %.

**Conclusions:**

New insecticides with different modes of action to those currently used in mosquito vector control are urgently needed. Indoxacarb shows great promise as a mixture with a pyrethroid and should be evaluated in experimental hut trials to determine performance against wild free-flying, pyrethroid resistant *An. gambiae* and wash-resistant formulations developed.

## Background

Insecticide-treated nets (ITN) are highly effective at reducing child mortality and incidence of uncomplicated and severe malaria [1]. Universal coverage with long-lasting insecticidal nets (LLINs) or indoor residual spraying (IRS) is a fundamental target for the protection of all people at risk of contracting malaria [2]. Between 2012 and 2014 (3 years), a cumulative total of 427 million LLINs were supplied for use in sub-Saharan Africa, mostly free of charge through mass distribution campaigns [3]. The rapid scale up of LLIN distribution has resulted in an estimated 49 % (44–54 %) of households in sub-Saharan Africa owning at least one ITN in 2013 compared with only 3 % in 2004 [3]. Since the launch of the US President’s Malaria Initiative in 2005, there has also been a substantial increase in IRS coverage in sub-Saharan Africa, with a peak of 77 million people (11 % of the at risk population) protected by IRS in 2011 [4]. Recently there has been a decrease in IRS coverage by 29 %, with only 55 million people protected in 2013 [3].

Pyrethroid insecticides are currently the only insecticides that are recommended by the WHO for use on LLINs [5]. Pyrethroids have been the chemical of choice for malaria vector control in recent decades but use of pyrethroids in agriculture and scaling up of malaria vector control has resulted in the evolution and spread of pyrethroid resistance in *Anopheles gambiae* sensu lato [6–8]. Target site insensitivity and metabolic resistance mechanisms are now widespread across sub-Saharan Africa and the effectiveness of LLINs and IRS treatment of houses with pyrethroid formulations is under threat [6, 9–11]. IRS formulations of insecticides with a different mode of action to pyrethroids are more costly, which has led to decreasing IRS coverage [12]. The situation for LLIN is more perilous, with no alternative insecticides currently recommended by WHO Pesticide Evaluation Scheme (WHOPES) for use on mosquito nets [5]. Cost-effective, safe insecticides with different modes of action to those currently used in public health are urgently needed to sustain the effectiveness of LLINs [13] and Innovative Vector Control Consortium (IVCC) are working together with industry and research partners to develop new active ingredients for vector control [14].

Indoxacarb was the first commercialized insecticide of a new class known as the oxadiazines and is highly efficacious against a wide range of agricultural pests [15] through a novel mode of action [16]. Indoxacarb was registered in 2000 by the United States Environmental Protection Agency (US EPA) in water dispersible granules (WG) (Avaunt^®^) and emulsifiable concentrate (EC) (Steward^®^) formulations, initially for foliar application targeting lepidopteran pests of cotton, rice, apples, pears, sweet corn, lettuce and fruiting vegetables [16, 17]. Indoxacarb has more recently been shown to be effective in the control of cockroaches [18], fire ants [19], termites [20], fleas [21], and houseflies [22] and has been commercialized as a gel bait (Advion^®^).

Indoxacarb is a neurotoxic insecticide that blocks voltage-dependent sodium channels, resulting in insect paralysis and death [15]. Despite the sodium channel being a well known target site for DDT and pyrethroids, crucially the mode of action for indoxacarb is distinct from other sodium channel targets [23]. This is possible due to the sodium channel being structurally large and complex, with at least 9 independent target sites for a variety of neurotoxins [24]. Indoxacarb is a pro-insecticide which is metabolized into the more active form after entering the insect host [16]. Bioactivation of indoxacarb (DPX-JW062) through decarbomethoxylation to the more active metabolite (DCJW) is attributed to the action of esterase and amidase enzymes within the insect [15, 24]. The active metabolite of indoxacarb exerts its effect by blocking the voltage-gated sodium ion channels in insects and is at least forty times more potent than parent indoxacarb in its ability to block sodium channel ion current [15, 21, 24].

Indoxacarb has proven effective as a broad spectrum oral insecticide against a wide variety of agricultural pests [17] but few studies have evaluated indoxacarb as a contact insecticide for vector control. Testing of indoxacarb treated polyester netting in Benin using WHO standard three minutes cone bioassay against *An. gambiae* showed a positive mortality dose–response, with dosages >100 mg/m^2^ producing mortality above the WHOPES threshold of 80 % [25, 26]. Tunnel test simulators using host-seeking mosquitoes confirmed good efficacy in terms of mortality at dosages >100 mg/m^2^, but there was no protection in terms of blood-feeding inhibition [25]. Time to first take-off testing demonstrated that even at a high dosage of 500 mg/m^2^ indoxacarb was only a mild irritant to *An. gambiae* and probably explains the lack of blood-feeding inhibition in tunnel tests [25]. Of critical importance was the lack of cross-resistance through mechanisms offering resistance to pyrethroids; no difference was found between the mortality rates for susceptible and pyrethroid resistant strains of *An. gambiae* [25]. The different target site on the sodium channel means that cross-resistance to existing pyrethroid or DDT target site-based resistance mechanisms is unlikely [24]. Indeed, strains of important crop pests the fall armyworm, *Spodoptera frugiperda*, and the diamondback moth, *Plutella xylostella*, which exhibit high levels of resistance to pyrethroids, showed no cross-resistance to indoxacarb [27, 28].

A LLIN that reduces the longevity of *Anopheles* mosquitoes but does not protect from biting can be a successful strategy at high coverage rates through a mass insecticidal effect [29]. An alternative strategy is to combine a non-repellent insecticide (to provide high levels of mortality) in a mixture with a pyrethroid insecticide (to provide protection against blood-feeding through repellency) [30]. In this study, indoxacarb was tested in bioassays and tunnel simulators as a single treatment and in a mixture with the pyrethroid alphacypermethrin against *An. gambiae* and *Culex quinquefasciatus* (pyrethroid susceptible and resistant strains) to determine its performance in terms of mortality and blood-feeding inhibition.

## Methods

### Insecticide formulations

Bioassay testing was conducted in parallel at three Pan-African Malaria Vector Research Consortium (PAMVERC) trial sites in Moshi and Muheza, Tanzania, and in Cotonou, Benin, during the course of a project with DuPont and Innovative Vector Control Consortium (IVCC). One hundred denier polyester netting was treated at PAMVERC trial sites with indoxacarb suspension concentrate (SC) 14.5 % or alphacypermethrin SC 2 % (DuPont, Wilmington, DE, USA). A tank mix was prepared at the field stations to treat netting with a mixture of the two insecticides.

### Toxicology

Indoxacarb has an excellent safety profile for mammals and is classified by US-EPA as ‘reduced risk’ [17]. Indoxacarb has a WHO toxicological classification II (moderately hazardous), an LD50 oral toxicity in rats of 268 mg/kg body weight and is category 3 under the Globally Harmonized System of Classification (GHS) [31]. This category is the same as the pyrethroid insecticides permethrin and deltamethrin, which are the most commonly used insecticides for LLINs [31, 32]. Mammals exhibit minimal bioactivation of indoxacarb to the more active metabolite, and the active metabolite has much weaker potency against mammalian sodium channels than insect sodium channels and is extensively metabolized and eliminated in the urine [33]. Indoxacarb has no known adverse impact on beneficiary insects [17, 24].

### Efficacy of indoxacarb ITN against *Anopheles gambiae* and *Culex quinquefasciatus* in tunnel tests

The tunnel test is a standard WHOPES specified laboratory system designed to allow expression of behavioural interactions that occur between free-flying mosquitoes and ITNs. Tunnel tests were carried out as a forerunner to experimental hut trials, as they provide information on dosage-dependent repellency, blood-feeding inhibition, mortality, and insecticide mixture interactions. Tunnel dimensions and protocols were as described in WHOPES guidelines for evaluation of ITN [34]. In one of the chambers, a guinea pig was housed unconstrained in a small wooden cage, and in the other chamber 50 unfed adult female *An. gambiae* of the pyrethroid susceptible Kisumu strain, aged 3–5 days, were released at dusk and left overnight. The netting surface was 400 cm^2^ and nine 1-cm diameter holes were cut into it (one hole located at the centre of the square, and the other eight holes equidistant and located 5 cm from the border) to give opportunity for mosquitoes to pass into the baited chamber. The following morning, live mosquitoes were removed from the chambers and held in paper cups under controlled conditions (25–27 °C and 75–85 % RH, with access to sugar solution), and monitored for delayed mortality for an additional 72 h after removal from the chamber.

In the first experiment netting samples treated with indoxacarb at dosages of 100, 500, and 1000 mg/m^2^ were tested against the pyrethroid susceptible strain *An. gambiae* Kisumu. Three replicates of 50 (n = 150) *An. gambiae* Kisumu mosquitoes per treatment were tested compared to an untreated control. In the second experiment efficacy of netting samples treated with indoxacarb at 500 mg/m^2^ was compared with alphacypermethrin at 20 and 40 mg/m^2^, based on the WHOPES recommended dosage range [35], against pyrethroid susceptible *Cx. quinquefasciatus* TPRI and resistant *Cx. quinquefasciatus* Muheza strains. Three replicates of 50 females were conducted for each treatment (n = 150) and strain. The *Cx. quinquefasciatus* Muheza strain was originally sampled from coastal Tanzania and colonized in the early 1990s but have since been selected with permethrin at the larval stage and is highly pyrethroid resistant in WHO cylinder tests (0.05 % permethrin papers, mortality = 15 %, n = 200). The use of the synergists PBO and DEF in bottle bioassays has demonstrated the presence of metabolic mechanisms of resistance in this strain (Matowo, personal communication).

### Efficacy of ITN mixture (indoxacarb + pyrethroid) against pyrethroid resistant *Cx. quinquefasciatus* in tunnel tests and bioassays

Efficacy of single treatments and mixtures of indoxacarb 25–300 mg/m^2^ and alphacypermethrin 25 mg/m^2^ was determined in tunnel tests. Two replicates of 50 (n = 100) pyrethroid resistant *Cx. quinquefasciatus* Cotonou strain were tested per treatment. In addition, a standard 3-min WHO wire-ball bioassay was conducted on the same netting samples with 4 replicates of 10 mosquitoes per sample (n = 40).

### Ethical approval

Ethical approval was granted from the Tanzania National Institute of Medical Research (NIMR/HQ/R.8c/Vol.I/24) and London School of Hygiene and Tropical Medicine Ethics Committee (Application no. 5162).

## Results

### Efficacy of indoxacarb ITN against *Anopheles gambiae* and *Culex quinquefasciatus* in tunnel tests

Mosquito netting treated with 500 or 1000 mg/m^2^ indoxacarb exceeded the WHOPES threshold of 80 % mortality (after 72 h) in tunnel tests (Table 1). Mortality rates were relatively low immediately after overnight exposure and there was delayed mortality up to 72 h after exposure. Unfed mosquitoes accounted for 96 % of the mortality that did occur immediately after overnight exposure across all dosages of indoxacarb. Treatment of netting with indoxacarb (100–1000 mg/m^2^) reduced mosquito penetration by approximately 40 % regardless of dosage (P > 0.05), and conferred protection by inhibiting between 60 and 75 % of blood-feeding (Table 1).

**Table 1:** Dosage testing of indoxacarb treated mosquito netting in tunnel tests against *Anopheles gambiae* Kisumu

Insecticide (mg/m^2^)	Mortality (%)				Penetration (%)	Blood-feeding (%)	Blood-feeding inhibition (%)
	Immediate	24 h	48 h	72 h			
Untreated	7^a^	7^a^	7^a^	7^a^	78^a^	73^a^	NA
Indoxacarb 100	46^b^	55^b^	60^b^	65^b^	48^b^	26^bc^	64
Indoxacarb 500	31^c^	68^c^	79^c^	85^c^	47^b^	18^b^	75
Indoxacarb 1000	45^b^	73^c^	86^c^	91^c^	51^b^	29^c^	60

If the superscript is the same in a column there was no significant difference between treatments

Alphacypermethrin-treated ITNs at 40 mg/m^2^ killed a far greater proportion of pyrethroid susceptible (78 % mortality) than resistant (21 % mortality) *Cx. quinquefasciatus* (P < 0.001) (Table 2). Indoxacarb-treated ITN (500 mg/m^2^) was equally effective against pyrethroid susceptible (67 % mortality) and resistant *Cx. quinquefasciatus* (75 % mortality) (P = 0.092). Indoxacarb (75 % mortality) was far more effective at killing pyrethroid resistant *Cx. quinquefasciatus* than was alphacypermethrin 40 mg/m^2^ (21 % mortality) (P < 0.001). Indoxacarb ITN (500 mg/m^2^) reduced blood-feeding by 100 and 76 % against the TPRI and Muheza strains respectively and equalled the performance of alphacypermethrin 20 mg/m^2^ (P > 0.05).

**Table 2:** Comparison of indoxacarb and pyrethroid treated mosquito netting in tunnel tests against pyrethroid susceptible and resistant *Culex quinquefasciatus*

Insecticide (mg/m^2^)	Mortality (%)				Penetration (%)	Blood-feeding (%)	Blood-feeding inhibition (%)
	Immediate	24 h	48 h	72 h			
*Culex quinquefasciatus* TPRI (pyrethroid susceptible)							
Untreated	0^a^	1^a^	8^a^	11^a^	57^a^	53^a^	NA
Alphacypermethrin 20	50^b^	52^b^	59^b^	59^b^	13^b^	1^b^	98
Alphacypermethrin 40	68^c^	69^c^	78^c^	78^c^	12^b^	0^b^	100
Indoxacarb 500	42^b^	54^b^	63^b^	67^b^	3^c^	0^b^	100
*Culex quinquefasciatus* Muheza (pyrethroid resistant)							
Untreated	0^a^	2^a^	3^a^	4^a^	67^a^	62^a^	NA
Alphacypermethrin 20	11^b^	22^b^	27^b^	29^b^	34^b^	17^b^	73
Alphacypermethrin 40	8^b^	17^b^	19^b^	21^c^	7^c^	4^c^	94
Indoxacarb 500	8^b^	58^c^	66^c^	75^d^	25^d^	15^b^	76

If the superscript is the same in a column there was no significant difference between treatments (analysed separately by species)

### Efficacy of a mixture of indoxacarb and pyrethroid applied to netting against pyrethroid resistant *Culex quinquefasciatus* Cotonou in tunnel tests and bioassays

**Fig. 1:**
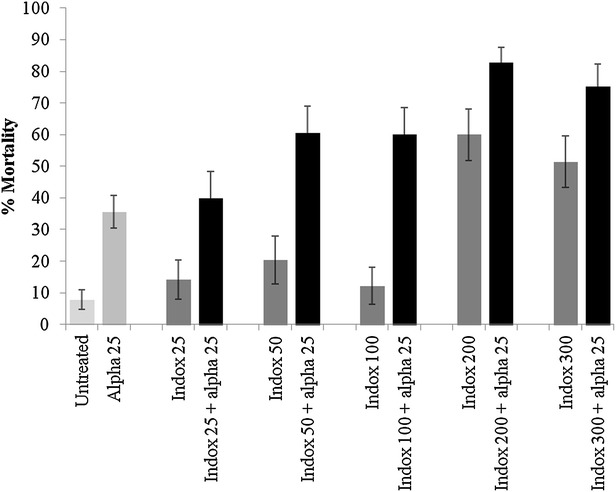
Percentage mortality of pyrethroid resistant *Cx. quinquefasciatus* Cotonou from tunnel tests with ITN samples of indoxacarb (indox) + alphacypermethrin (alpha) mixture compared with single treatments

**Fig. 2:**
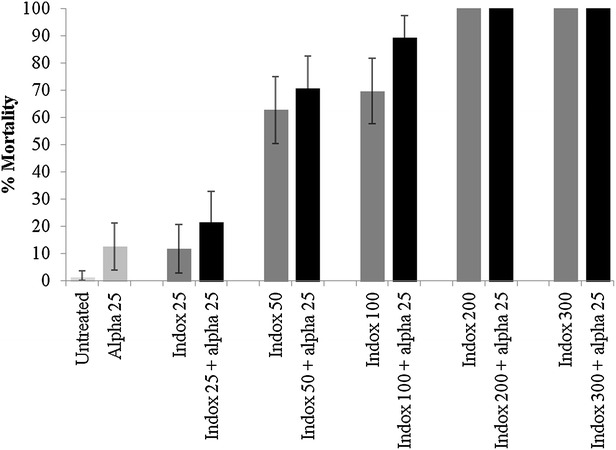
Percentage mortality of pyrethroid resistant *Cx. quinquefasciatus* Cotonou from ball-frame bioassays with a mixture of indoxacarb + alphacypermethrin compared with single treatments

**Fig. 3:**
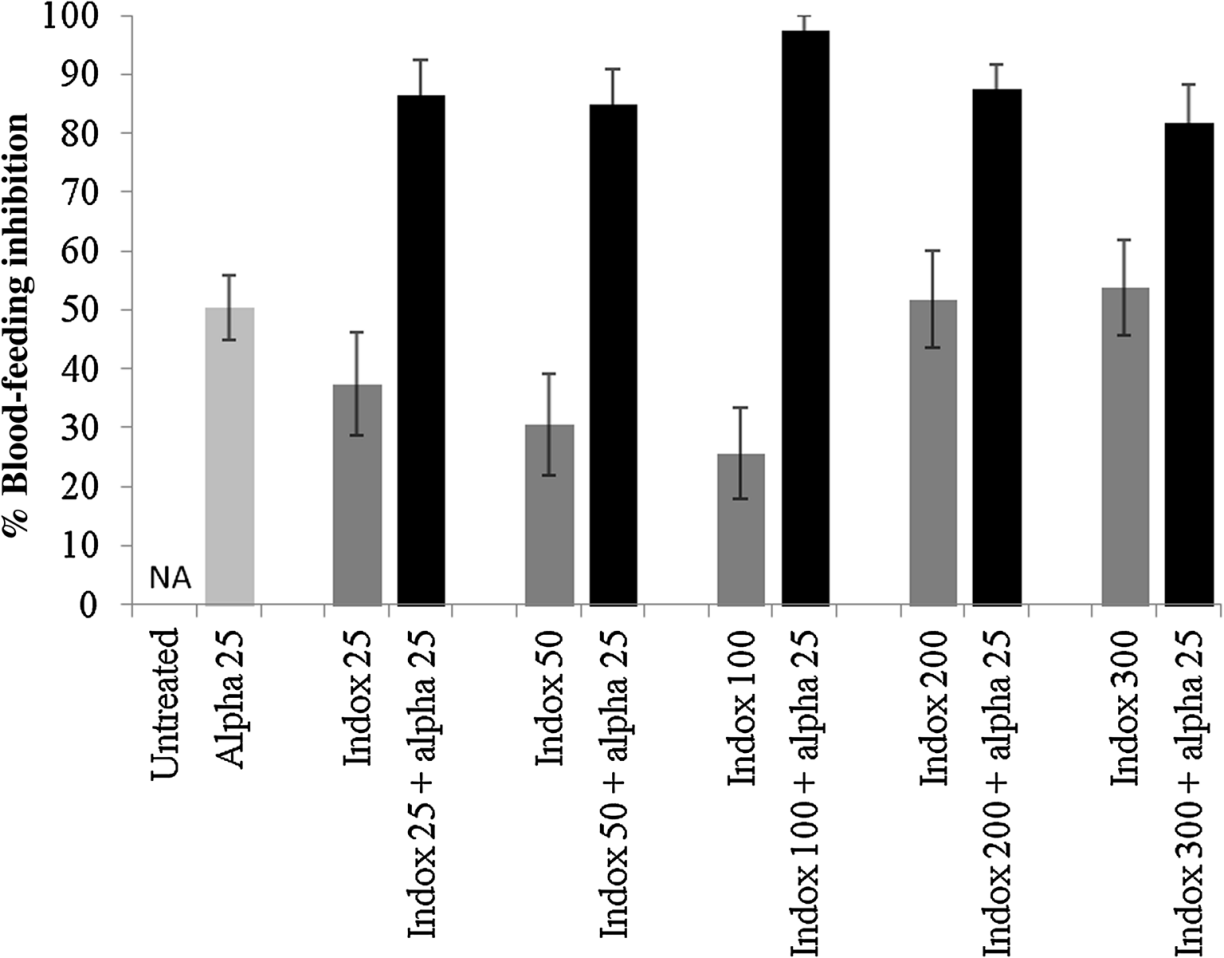
Comparison of netting treated with a mixture of indoxacarb + alphacypermethrin with single treatments in tunnel tests in terms of blood-feeding inhibition against pyrethroid resistant *Cx. quinquefasciatus* Cotonou strain

Netting treated with alphacypermethrin at 25 mg/m^2^ killed only 35 % of pyrethroid resistant *Cx. quinquefasciatus* Cotonou in tunnel tests (Fig. 1). Indoxacarb ITN at low dosages of 25–100 mg/m^2^ produced low levels of mortality (<20 %) but indoxacarb ITN at 200 and 300 mg/m^2^ killed 60 and 52 % of mosquitoes respectively. Mixtures always killed a higher proportion than the single treatments, with indoxacarb 200 mg/m^2^ + alphacypermethrin 25 mg/m^2^ killing the highest proportion of mosquitoes (83 % mortality) (Fig. 1). Ball-frame bioassays using the same netting pieces produced 100 % mortality for indoxacarb samples at 200 and 300 mg/m^2^ but only 13 % mortality for the pyrethroid (Fig. 2). In tunnel tests, the pyrethroid reduced blood-feeding by 50 % compared to the untreated control (Fig. 3). Indoxacarb at 200 and 300 mg/m^2^ produced equivalent feeding inhibition at 52 and 54 % respectively (P > 0.05). Lower dosages of indoxacarb 25–100 mg/m^2^ were less effective than alphacypermethrin (P < 0.05) and reduced blood-feeding by between 26 and 40 %. Mixtures conferred far greater protection than the single treatments, with all dosages producing levels of blood-feeding inhibition >80 % (Fig. 3).

## Discussion

Pyrethroid resistance has become widespread in malaria vectors throughout sub-Saharan Africa and the lack of alternative insecticides for use on mosquito nets is a particularly serious threat to malaria vector control [5, 6]. The Global Plan for Insecticide Resistance Management in Malaria Vectors (GPIRM) states that if pyrethroids were to lose most of their efficacy 55 % of the benefits of vector control would be lost, leading to approximately 120,000 deaths not averted [36]. New insecticides for ITN should ideally reduce the mean life expectancy of *Anopheles* mosquitoes through a mass killing, provide individual user protection through repellency, show no cross-resistance to existing insecticides used for malaria control and be safe for humans and non-target organisms [14, 36]. Standard WHOPES 3 min ball-frame bioassay produced 100 % mortality (72 h) for dosages of indoxacarb >100 mg/m^2^, which is well above the threshold of 80 % specified in WHO guidelines for evaluation of LLINs [26]. Similarly, indoxacarb produced high levels of mortality in tunnel tests against both *An. gambiae* and *Cx. quinquefasciatus* at dosages >100 mg/m^2^. Although the majority of mortality occurred within 24 h of testing, delayed mortality should routinely be recorded up to 72 h after exposure. Insect species metabolize indoxacarb rapidly to the more active metabolite DCJW after ingestion, but more slowly after topical treatment, thus explaining the delayed mortality [15]. Indoxacarb is conventionally thought to produce relatively low levels of repellency against mosquitoes [25] and agricultural pests [20], however in these studies high levels of blood-feeding inhibition were achieved with *An. gambiae* and *Cx. quinquefasciatus*, particularly at dosages >100 mg/m^2^. Reduced penetration of holed, indoxacarb treated netting may be indicative of indoxacarb-induced repellency. However, 96 % of *An. gambiae* mosquitoes killed immediately after overnight exposure were unfed, indicating that these were killed rapidly before being able to penetrate the netting and blood-feed. Mixtures of indoxacarb and alphacypermethrin produced particularly impressive levels of blood-feeding inhibition, with an apparent additive effect.

As with any new insecticide, questions must be asked regarding whether cross-resistance might be conferred by mechanisms of insecticide resistance that have developed in field populations of the target species, how rapidly resistance might develop, and which genes might be involved [36]. Crucially, no cross-resistance has been detected to indoxacarb in a pyrethroid resistant strain of *Cx. quinquefasciatus* or in pyrethroid resistant strains of agricultural pests [22, 27, 28]. In houseflies, two insectary strains 5900 and 18,000 fold resistant to permethrin showed no cross-resistance to indoxacarb despite the presence of kdr and increased oxidative metabolism mediated by cytochrome P450 CYP6D1 [22]. The lack of cross-resistance is partly due to the distinctive target site on the sodium channel compared to other insecticides that target sodium channels such as pyrethroids and DDT [24]. The only record of indoxacarb resistance in wild mosquito populations was reported for *Aedes albopictus* in Pakistan and was attributed to intensive indoxacarb application for the control of cotton pests over several generations and not due to cross-resistance [37]. Indoxacarb has a solubility in water of 0.20 mg/l at 25 °C which is similar to alphacyano-pyrethroids and pyrrole insecticides [38]. As with these other classes of insecticide with low water solubility, development of wash resistant formulations for long lasting treatment of mosquito nets should be feasible.

## Conclusions

With the development and spread of resistance to pyrethroids, carbamates, and organophosphate insecticides, new insecticides with different modes of action to those currently used are urgently needed if effective malaria vector control is to be sustained. In this study tunnel tests and bioassays have shown that indoxacarb is highly effective both in terms of mortality against a pyrethroid susceptible strain of *An. gambiae* and against pyrethroid susceptible and resistant strains of *Cx. quinquefasciatus* and in reducing the amount of blood-feeding by these mosquito strains. Indoxacarb appears to be particularly promising when deployed as a mixture with a pyrethroid and should be evaluated in experimental hut trials against wild free-flying, pyrethroid resistant *An. gambiae* and with wash-resistant formulations.

## References

[1] Lengeler C. Insecticide-treated bed nets and curtains for preventing malaria. Cochrane Database Syst Rev 2004; (2) CD000363. 10.1002/14651858.CD000363.pub2 15106149

[2] RBM. Malaria implementation guidance in support of the preparation of concept notes for the Global Fund. Roll Back Malaria Harmonization Working Group. 2014.

[3] WHO (2014). World Malaria Report 2014.

[4] WHO (2012). World Malaria Report 2012.

[5] WHOPES (2012). WHO recommended long-lasting insecticidal mosquito nets.

[6] Ranson H, N’Guessan R, Lines J, Moiroux N, Nkuni Z, Corbel V (2011). Pyrethroid resistance in African anopheline mosquitoes: what are the implications for malaria control?. Trends Parasitol.

[7] Diabate A, Baldet T, Chandre F, Akogbeto M, Guiguemde TR, Darriet F (2002). The role of agricultural use of insecticides in resistance to pyrethroids in *Anopheles gambiae* s.l. in Burkina Faso. Am J Trop Med Hyg.

[8] Czeher C, Labbo R, Arzika I, Duchemin JB (2008). Evidence of increasing Leu-Phe knockdown resistance mutation in *Anopheles gambiae* from Niger following a nationwide long-lasting insecticide-treated nets implementation. Malar J.

[9] N’Guessan R, Corbel V, Akogbeto M, Rowland M (2007). Reduced efficacy of insecticide-treated nets and indoor residual spraying for malaria control in pyrethroid resistance area, Benin. Emerg Infect Dis.

[10] Corbel V, Akogbeto M, Damien GB, Djenontin A, Chandre F, Rogier C (2012). Combination of malaria vector control interventions in pyrethroid resistance area in Benin: a cluster randomised controlled trial. Lancet Infect Dis.

[11] Sharp BL, Ridl FC, Govender D, Kuklinski J, Kleinschmidt I (2007). Malaria vector control by indoor residual insecticide spraying on the tropical island of Bioko, Equatorial Guinea. Malar J.

[12] President’s Malaria Initiative: President’s Malaria Initiative Ethiopia Malaria Operational Plan FY 2015. 2015.

[13] Zaim M, Guillet P (2002). Alternative insecticides: an urgent need. Trends Parasitol.

[14] Hemingway J, Beaty BJ, Rowland M, Scott TW, Sharp BL (2006). The innovative vector control consortium: improved control of mosquito-borne diseases. Trends Parasitol.

[15] Wing KD, Sacher M, Kagaya Y, Tsurubuchi Y, Mulderig L, Connair M (2000). Bioactivation and mode of action of the oxadiazine indoxacarb in insects. Crop Protection.

[16] McCann SF, Annis GD, Shapiro R, Piotrowski DW, Lahm GP, Long JK (2001). The discovery of indoxacarb: oxadiazines as a new class of pyrazoline-type insecticides. Pest Manag Sci.

[17] EPA US. Indoxacarb conditional registration. EPA fact sheet 2000.

[18] Anikwe JC, Adetoro FA, Anogwih JA, Makanjuola WA, Kemabonta KA, Akinwande KL (2014). Laboratory and field evaluation of an indoxacarb gel bait against two cockroach species (Dictyoptera: Blattellidae, Blattidae) in Lagos, Nigeria. J Econ Entomol.

[19] Oi DH, Oi FM (2006). Speed of efficacy and delayed toxicity characteristics of fast-acting fire ant (Hymenoptera: Formicidae) baits. J Econ Entomol.

[20] Hu XP (2005). Evaluation of efficacy and nonrepellency of indoxacarb and fipronil-treated soil at various concentrations and thicknesses against two subterranean termites (Isoptera: Rhinotermitidae). J Econ Entomol.

[21] Dryden MW, Payne PA, Smith V, Heaney K, Sun F (2013). Efficacy of indoxacarb applied to cats against the adult cat flea, *Ctenocephalides felis*, flea eggs and adult flea emergence. Parasit Vectors.

[22] Shono T, Zhang L, Scott JG (2004). Indoxacarb resistance in the house fly, *Musca domestica*. Pest Biochem Physiol.

[23] Scott JG (1988). Pyrethroid insecticides. ISI Atlas Sci Pharmacol.

[24] Silver KS, Song W, Nomura Y, Salgado VL, Dong K (2010). Mechanism of action of sodium channel blocker insecticides (SCBIs) on insect sodium channels. Pestic Biochem Physiol.

[25] N’Guessan R, Corbel V, Bonnet J, Yates A, Asidi A, Boko P, Odjo A, Akogbeto M, Rowland M (2007). Evaluation of indoxacarb, an oxadiazine insecticide for the control of pyrethroid-resistant *Anopheles gambiae* (Diptera: Culicidae). J Med Entomol.

[26] WHO. Guidelines for laboratory and field-testing of long-lasting insecticidal nets. WHO/HTM/NTD/WHOPES/2013.1 .

[27] Yu SJ, McCord E (2007). Lack of cross-resistance to indoxacarb in insecticide-resistant *Spodoptera frugiperda* (Lepidoptera: Noctuidae) and Plutella xylostella (Lepidoptera: Yponomeutidae). Pest Manag Sci.

[28] Sayyed AH, Ahmad M, Saleem MA (2008). Cross-resistance and genetics of resistance to indoxacarb in *Spodoptera litura* (Lepidoptera: Noctuidae). J Econ Entomol.

[29] Massad E, Coutinho FA (2012). Vectorial capacity, basic reproduction number, force of infection and all that: formal notation to complete and adjust their classical concepts and equations. Mem Inst Oswaldo Cruz.

[30] N’Guessan R, Ngufor C, Kudom AA, Boko P, Odjo A, Malone D (2014). Mosquito nets treated with a mixture of chlorfenapyr and alphacypermethrin control pyrethroid resistant *Anopheles gambiae* and *Culex quinquefasciatus* mosquitoes in West Africa. PLoS One.

[31] WHO (2009). Recommended classification of pesticides by hazard and guidelines to classification.

[32] van den Berg H, Zaim M, Yadav R, Soares A, Ameneshewa B, Mnzava AE (2012). Global trends in the use of insecticides for vector-borne disease control. Environ Health Pers.

[33] Dias JL. Enviromental fate of indoxacarb. Dept Pest Regist 2006. http://www.cdpr.ca.gov/docs/emon/pubs/fatememo/indoxupdate.pdf. Accessed 11 Feb 2015

[34] WHO. Guidelines for testing mosquito adulticides for indoor residual spraying and treatment of mosquito nets. WHO/CDS/NTD/WHOPES/GCDPP/2006.3.

[35] WHO (2007). Recommended insecticide products treatment of mosquito nets for malaria vector control.

[36] WHO (2012). The global plan for insecticide resistance management in malaria vectors (GPIRM).

[37] Khan HA, Akram W, Shehzad K, Shaalan EA (2011). First report of field evolved resistance to agrochemicals in dengue mosquito, *Aedes albopictus* (Diptera: Culicidae), from Pakistan. Parasit Vectors.

[38] MacBean C. The pesticide manual : a world compendium. In: MacBean C (ed). 16th edn. Alton: BCPC; 2012.

